# Contact Effect of a *Methylobacterium* *sp*. Extract on Biofilm of a *Mycobacterium* *chimaera* Strain Isolated from a 3T Heater-Cooler System

**DOI:** 10.3390/antibiotics9080474

**Published:** 2020-08-03

**Authors:** Inés Pradal, Jaime Esteban, Arancha Mediero, Marta García-Coca, John Jairo Aguilera-Correa

**Affiliations:** 1 Clinical Microbiology Department, IIS-Fundación Jiménez Díaz, UAM, 28040 Madrid, Spain; inespradalap@gmail.com (I.P.); john_j2a@hotmail.com (J.J.A.-C.); 2 Bone and Joint Unit, IIS-Fundación Jiménez Díaz, UAM, 28040 Madrid, Spain; aranzazu.mediero@quironsalud.es; 3 Clinical Microbiology Department, Quironsalud-Madrid University Hospital, 28223 Pozuelo de Alarcón, Spain; marta.gcoca@quironsalud.es

**Keywords:** *Mycobacterium chimaera*, 3T HCD, biofilm, *Methylobacterium*, antibiofilm effect

## Abstract

*Mycobacterium chimaera* is an opportunistic slowly growing non-tuberculous mycobacteriumof increasing importance due to the outbreak of cases associated with contaminated 3T heater-cooler device (HCD) extracorporeal membrane oxygenator (ECMO). The aim of this study was to evaluate the effect of pre-treating a surface with a *Methylobacterium sp.* CECT 7180 extract to inhibit the *M. chimaera* ECMO biofilm as well as of the treatment after different dehydration times. Surface adherence, biofilm formation and treatment effect were evaluated by estimating colony-forming units (CFU) per square centimeter and characterizing the amount of covered surface area, thickness, cell viability, and presence of intrinsic autofluorescence at different times using confocal laser scanning microscopy and image analysis. We found that exposing a surface to the *Methylobacterium sp.* CECT 7180 extract inhibited *M. chimaera* ECMO biofilm development. This effect could be result of the effect of *Methylobacterium* proteins, such as DNaK, trigger factor, and xanthine oxidase. In conclusion, exposing a surface to the *Methylobacterium* *sp*. extract inhibits *M. chimaera* ECMO biofilm development. Furthermore, this extract could be used as a pre-treatment prior to disinfection protocols for equipment contaminated with mycobacteria after dehydration for at least 96 h.

## 1. Introduction

*Mycobacterium chimaera* is a strictly aerobic, pleomorphic, non-motile and non-spore forming [1], slowly growing non-tuberculous mycobacterium mainly acting as an opportunistic pathogen [2,3,4]. *M. chimaera* has been found in several environments such as soil, plants, animals, and especially in water distribution systems. Contagion takes place via natural reservoirs, but never from human to human. Most documented cases of *M. chimaera* have taken place in Europe and Unites States, where its prevalence is increasing, mostly among immunocompromised patients and individuals with indwelling prosthetic devices [4,5,6].

The increasing importance of this mycobacterium stems from the numerous cases of disseminated infection associated with the use of 3T HCD extracorporeal oxygenator (LivaNova) [7], an essential medical device employed in open-chest surgeries and one of the best sellers in the world, with 80% of the devices in commerce [8]. Since 2011, when the first case appeared in Munich (Germany), many similar cases have been reported around the world: in Europe (30 cases in United Kingdom, nine in Italy, five in Germany, four in The Netherlands and Ireland, three in France, andone in Spain), in America (18 in United States and, two in Canada), in Asia (at least one case in China), and in Oceania (15 cases in Australia and New Zeeland) [9,10,11,12,13,14,15,16,17,18,19]. Indeed, taking into account that not all the hospital cases are reported in scientific articles, the actual prevalence of *M. chimaera* infections related to the use of 3T HCD extracorporeal could range between 156 and 282 cases in Europe alone [20]. Each case showed a similar past history and symptoms: all patients underwent open-chest surgery, and the main manifestation was prosthetic valve endocarditis [21,22]. Moreover, other clinic manifestations have been described associated to this infection, such as osteoarthritis, spondylodiscitis, breastbone infections, hepatitis, nephritis, and bacteremia [23]. However, there can be between five and 38 months after the cardiac surgery before one can detect any of these symptoms [9]. Noteworthy, *M. chimaera* is characterized by showing resistance to a great number of antimycobacterial antibiotic and by requiring a treatment from three to six months and at least six months more after a negative sputum culture [3,24]. For that, the prognosis associated with these infections is usually poor and has reached a mortality rate of 50% in United States [6]. 

All isolated strains from these above-mentioned cases were genetically related and were found in the oxygenation membrane, indicating that this outbreak was an origin contamination, meaning it originated in the factory [25,26]. In-depth studies have shown that the infection focus was *M. chimaera* biofilms on the membrane of the 3T HCD extracorporeal oxygenators, a membrane placed between two water tanks used to maintain blood and cardioplegia solution at a constant temperature and control oxygen level in patient blood [21,22]. Thereupon, *M. chimaera* would reach the bloodstream due to the formation and dispersion of aerosols harboring small pieces of *M. chimaera* biofilm from the membrane [21,22]. Following these reports, the manufacturer of the device recalled a part of the instruments and recommended to use a stricter and deeper disinfection [27] and new revised protocols of use [13,27,28,29] which turned out to be completely ineffective [30]. Indeed, Food and Drug Administration (FDA) and American and European Control Disease Centers have issued alerts about these disinfections and keep on still monitoring the appearance of new cases [10,31]. Thus, to establish new decontamination and treatment strategies in the future, antibiofilm compounds against mycobacteria need to be studied in depth.

A new mycobacterial antibiofilm strategy based on the use of *Methylobacterium spp.* is currently being developed [32,33]. *Methylobacterium* is a genus from Alphaproteobacteria isolated by Patt et al. [34] that is a strictly aerobic bacterium and forms small rose colonies at its optimal growth temperature (25 °C) and usually inhabits water distribution systems [32,35]. Some strains of *Methylobacterium* genus have already demonstrated antibacterial effect against different microorganisms, both Gram-positive and Gram-negative bacteria [36,37,38], including mycobacteria [33], and fungi [36].

Therefore, the aim of this study was to evaluate the contact effect of a *Methylobacterium sp.* extract to inhibit and treat the biofilm of a *M. chimaera* strain isolated from a 3T HCD extracorporeal oxygenator membrane.

## 2. Results

### 2.1. Methylobacterium sp. CECT 7180 Does Not Inhibit M. chimaera ECMO Adherence

**Figure 1:**
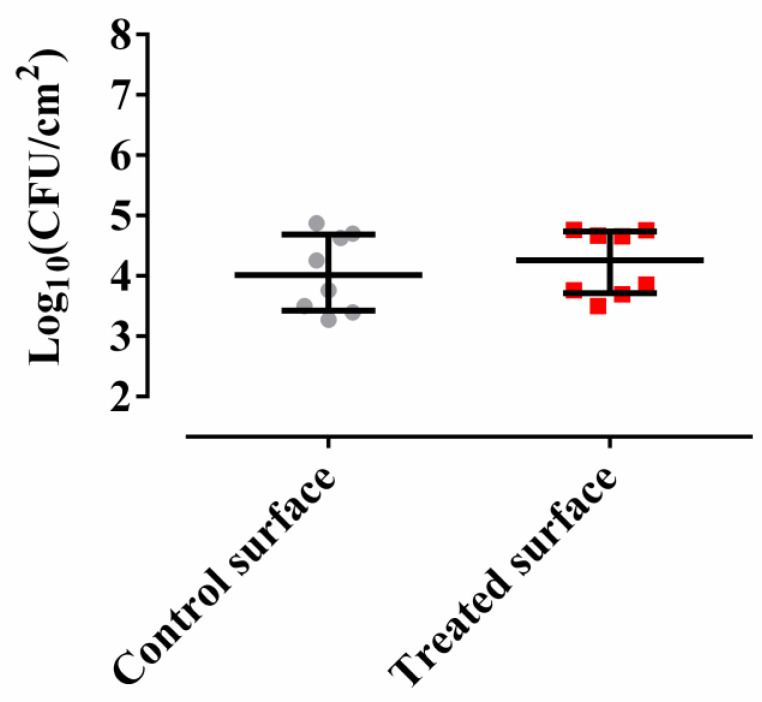
*M. chimaera* ECMO adherence to a control surface (gray) and a surface treated with *Methylobacterium sp.* CECT 7180 extract (red). The bars represent the interquartile range.

Non-significant differences were detected between *M. chimaera* ECMO adherence to the control surface and to a surface treated with the *Methylobacterium sp*. CECT 7180 extract (*p*-value = 0.8336 for Wilcoxon test) (Figure 1), indicating that the *Methylobacterium sp*. CECT 7180 extract has no anti-adherent effect on *M. chimaera* ECMO.

### 2.2. Methylobacterium sp. CECT 7180 Inhibits the Formation of M. chimaera ECMO Biofilm

**Figure 2:**
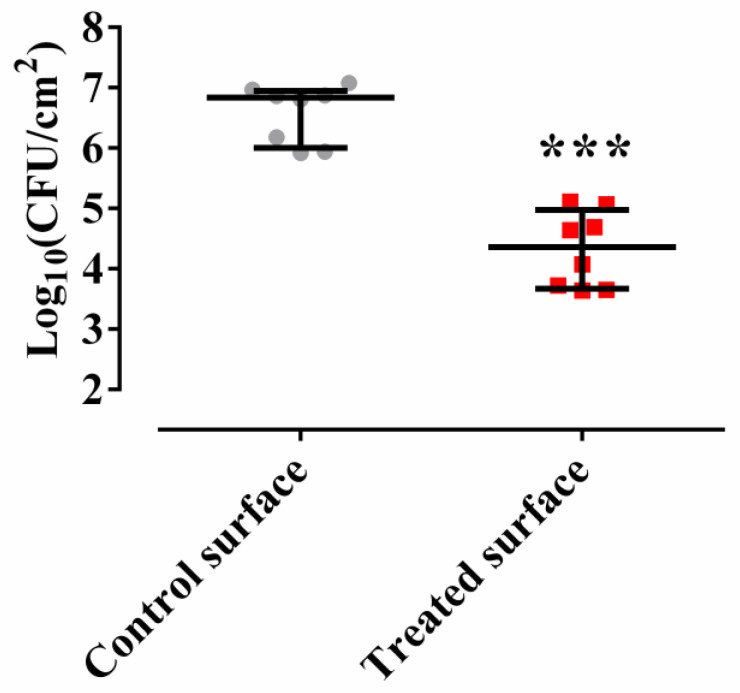
*M. chimaera* ECMO biofilm formation on a control surface (gray) or on a surface treated with *Methylobacterium sp*. CECT 7180 extract (red). The bars represent the interquartile range. *** *p*-value < 0.001 for Wilcoxon test.

The treatment of a surface with the *Methylobacterium sp*. CECT 7180 extract decreased biofilm formation. This could be observed macroscopically, and by comparing colony forming units per area count on a control surface, Log_10_(CFU/cm^2^) = 6.836, with those on a treated surface, Log_10_(CFU/cm^2^) = 4.357 (*p*-value = 0.0008 for Wilcoxon test) (Figure 2). It is noteworthy to highlight that the adhered colony forming units per area count on a treated surface did not change with the time, and there were no significative differences between the number of adhered bacteria on a treated surface after the 90 min of exposure (Log_10_(CFU/cm^2^) = 4.26) and the number of adhered bacteria on a treated surface and grown in Middlebrook for 120 h (Log_10_(CFU/cm^2^) = 4.357) in the same surface (*p*-value = 0.8541 for Wilcoxon test).

**Figure 3:**
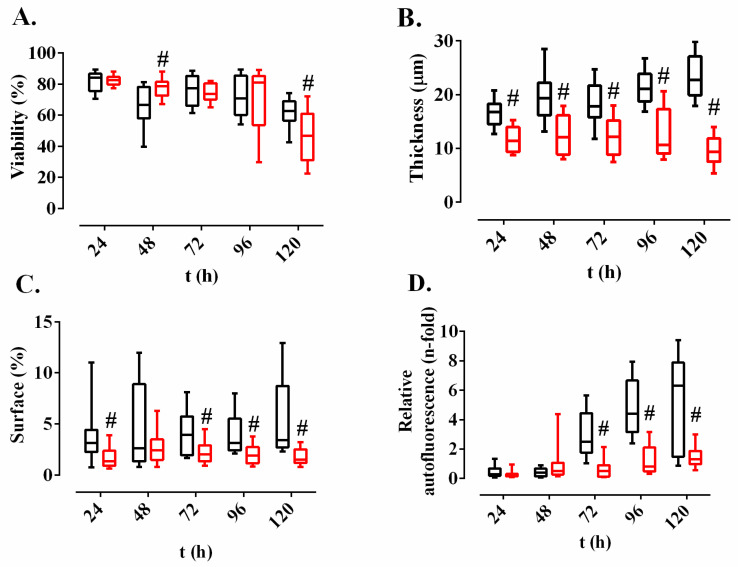
*M. chimaera* ECMO biofilm development over time on a control surface (black) and on a surface treated with *Methylobacterium* extract (red). The four parameters evaluated were (**A**) mycobacterial viability (%); (**B**) biofilm thickness (µm); (**C**) biofilm covered surface (%); and (**D**) relative autofluorescence (n-fold). The boxes represent the median and interquartile range and the bars indicate tenth and ninetieth percentiles. #: *p*-value < 0.001 for Wilcoxon test between on a control surface and on a surface treated with *Methylobacterium sp.* CECT 7180 extract.

The process of *M. chimaera* ECMO biofilm growth was characterized over 120 h by confocal laser microscopy on a control surface and on a surface that had been treated with *Methylobacterium sp*. CECT 7180 extract. The biofilm formation process on the control surface showed a slight reduction over time of the viable bacteria, since significant differences were found between 24 and 48 h (*p*-value < 0.0001 for Wilcoxon test), 48 and 72 h (*p*-value = 0.0057 for Wilcoxon test), and 96 and 120 h (*p*-value = 0.0101 for Wilcoxon test) (Figure 3A, in black). The biofilm grew in thickness over time (*p*-value < 0.0001 for Kruskal-Wallis test) (Figure 3B, in black) but did not change the covered surface (*p*-value = 0.3669 for Krustal-Wallis test) (Figure 3C, in black). Relative autofluorescence was similar between 24 and 48 h (*p*-value = 0.6789 for Wilcoxon test) and significantly increased from 72 to 120 h (*p*-value < 0.0001 for Wilcoxon test) (Figure 3D, in black).

The biofilm grown on the surface treated with *Methylobacterium* extract showed a slight but statistically significant reduction between 24 and 48 h (*p*-value = 0.0026 for Wilcoxon test), and a substantial decrease of the viable bacteria (%) between 96 and 120 h (*p*-value = 0.0001 for Wilcoxon test) (Figure 3A, in red). Thickness showed non-significant differences between 24 and 96 h (*p*-value = 0.9611 for Kruskal-Wallis test), but at 120 h it significantly decreased (*p*-value = 0.0223 for Wilcoxon) (Figure 3B, in red). However, covered surface increased between 24 and 48 h (*p*-value = 0.0163 for Wilcoxon test) and, from this time, there were no significant differences over time (Kruskal-Wallis *p*-value = 0.9611) (Figure 3C, in red). Finally, relative autofluorescence increased slightly but significantly between 24 and 48 h (*p*-value = 0.0004 for Wilcoxon test) and between 72 and 96 h (*p*-value = 0.0078 for Wilcoxon test) but showed no differences for other times (Figure 3D, in red).

**Figure 4:**
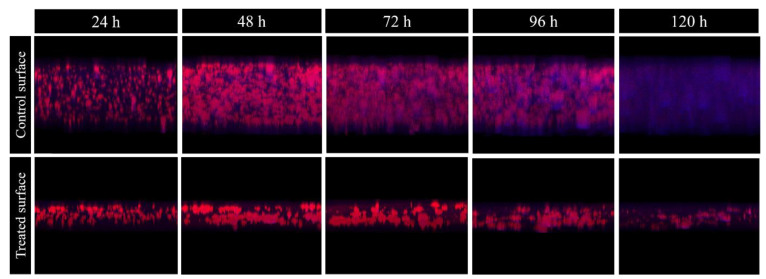
3D structure of *M. chimaera* ECMO biofilms of different ages (24, 48, 72, 96, and 120 h), grown on a control surface (control) or on a treated surface with *Methylobacterium sp.* CECT 7180 (Treated). In red, the red Nile stain which corresponds to the wall of mycobacteria forming the biofilm, and in blue, the relative autofluorescence, which would correspond to the biofilm matrix.

When both surface conditions were compared, the following differences were detected (Figure 3): thickness, surface covered, and relative autofluorescence were significantly lower in the biofilm grown on the surface treated with *Methylobacterium* extract. These differences can be also seen in the 3D reconstructions (Figure 4).

### 2.3. Effect of Methylobacterium sp. CECT 7180 Extract on M. chimaera ECMO Biofilms Dehydrated for Different Time Periods

**Figure 5:**
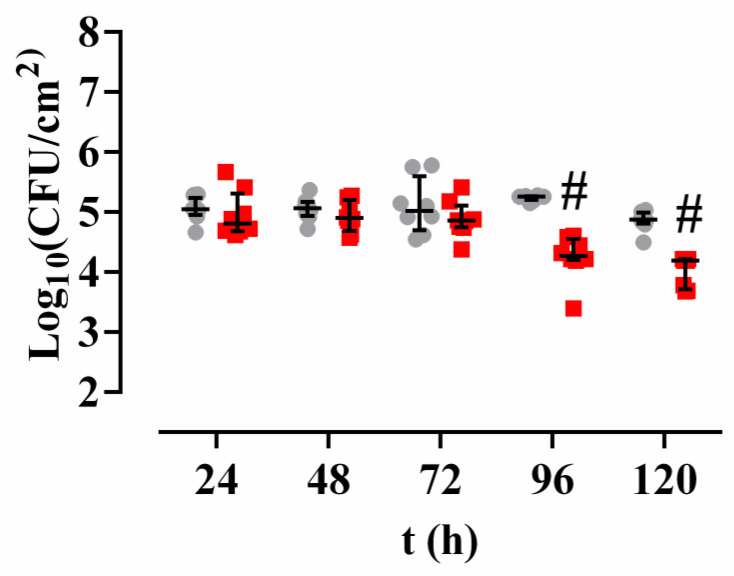
Amount of *M. chimaera* ECMO per area unit after different times of dehydration and treated (red) or not (gray) with *Methylobacterium* extract. The bars represent the interquartile range. #: *p*-value < 0.001 for Wilcoxon test between a control treatment or a treatment with *Methylobacterium sp*. CECT 7180 extract.

Dehydration from 24 to 96 h did not decrease the mycobacterial viability over time (*p*-value = 0.1239 for Kruskal-Wallis), but there was a significant decrease between 96 and 120 h (*p*-value = 0.0008 for Wilcoxon test) (Figure 5).

Treatment with *Methylobacterium* extract did not have any effect on biofilms dehydrated for 24, 48, or 72 h (Figure 5) (*p*-value = 0.5588 for Kruskal-Wallis test). However, treatment with the *Methylobacterium* extract provoked a significant drop in the viability of the biofilms dehydrated for 96 and for 120 h (Figure 5), therefore it can be said that the treatment with *Methylobacterium* extract was only effective after 96 and/or 120 h of desiccation at room temperature (*p*-value < 0.001).

### 2.4. Study of Adhered Proteins of Methylobacterium sp. CECT 7180 Extract

**Figure 6:**
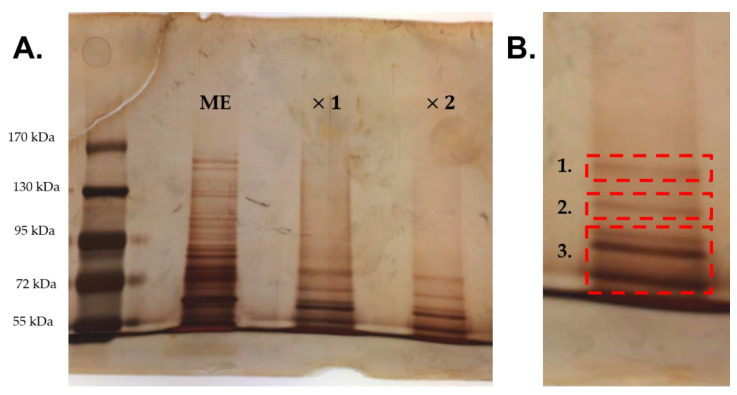
Silver stained protein band pattern of pure *Methylobacterium sp.* extract (ME) and detached proteins from *Methylobacterium sp.* extract adhesion washed once (×1) or twice (×2) separated by SDS Polyacrylamide Gel Electrophoresis (PAGE) (**A**); Bands selected for LC-MS/MS analysis (**B**).

With silver staining, at least three prominent single groups of bands were observed (Figure 6A). The three groups of bands were cut out in the gel from the detached proteins from *Methylobacterium spp.* extract adhesion washed twice, since it had less background (Figure 6B).

A total of 21 proteins were identified by Liquid Chromatography Electrospray Ionization Tandem Mass Spectrometric (LC ESI-MS/MS) in the bands cut out and are included in the Table 1. Six *Methylobacterium sp.* proteins showed more than one peptide and were considered predominant in each band (Table 1, in bold). In band 1, an elongation factor G was identified among other proteins. In band 2, a translation initiation factor 2, a molecular chaperone DnaK, a 30S ribosomal protein S1 and a xanthine oxidase family protein molybdopterin-binding subunit were identified among other proteins. In band 3, a trigger factor was mainly identified among other proteins.

## 3. Discussion

This study demonstrates that the *Methylobacterium sp*. extract can inhibit the *M. chimaera* ECMO biofilm development when it is used to treat a plastic surface. Further, we demonstrated a possible alternative method to decontaminate 3T HCD extracorporeal oxygenator membranes after allowing the oxygenators to dry at room temperature for at least four or five days.

As in many microorganisms, mycobacterial biofilm development starts with an adherence stage, and then proceeds to the maturation stage, which consists of sessile growth and matrix synthesis and, finally, the dispersion [32]. The *M. chimaera* ECMO biofilm formation process is highly similar to the constant mycobacterial viability of *M. mageritense* [39]. Interestingly, there was a slightly but statistically significant reduction of the viability in the control condition that might be due to a controlled autolysis which may justify the modest increase of the relative autofluorescence at 72 h to favor the biofilm development [40]. The *M. chimaera* ECMO biofilm formation process is also similar to the absence of surface growth over time of *M. mageritense*, the vertical growth of *Mycobacterium peregrinum*, and the autofluorescence increase from 24 to 48 h of *M. chelonae*, *M. peregrinum*, and *M. fortuitum* [39].

In our study, the effect of the *Methylobacterium* extract did not compromise *M. chimaera* adherence. By contrast, other *Methylobacterium* strains have been found to exert an anti-adherent effect on *Mycobacterium avium* [35]. According to our results, the action of the *Methylobacterium* extract adhered on a surface takes place after the mycobacterial adherence.

The thickness, surface coverage, and relative autofluorescence of the *M. chimaera* ECMO biofilm were significantly lower on surfaces treated with *Methylobacterium* extract. The most critical effect of the *Methylobacterium* extract was the significant inhibition of relative autofluorescence. Relative autofluorescence is mainly caused by F420 coenzyme presence in the mycobacterial wall and biofilm matrix [32]. Therefore, a decrease in relative autofluorescence, when the bacterial viability remains relatively constant over time, indicates a decrease in biofilm matrix synthesis. As biofilm formation is regulated by quorum-sensing [41], we hypothesize that this *Methylobacterium* extract may also exert a quorum quenching-like effect on *M. chimaera* ECMO. This antibiofilm effect has also been described in other rapidly growing non-tuberculous mycobacteria, such as *Mycobacterium abscessus*, *Mycobacterium fortuitum*, and *Mycobacterium chelonae* [35]. This antibacterial effect has been described in other *Methylobacterium* strains, for instance, the ERI-135 strain isolated from the soil whose ethyl acetate extract inhibited the growth of bacteria such as *Bacillus subtilis*, *Klebsiella pneumoniae*, *Pseudomonas aeruginosa*, *Salmonella typhimurium*, *Shigella flexneri*, *Enterobacter aerogenes*, *Staphylococcus aureus* and *S. epidermidis* and fungi, such as *Candida albicans* and *Trichophyton rubrum* [38]; the *Methylobacterium radiotolerans* MAMP 4754 isolated from the seeds of the river bushwillow (*Combretum erythrophyllum* (Burch.) Sond), whose ethyl acetate and chloroform extracts also showed antibacterial effect on *B. subtilis, B. cereus, Escherichia coli, K. oxytoca,* and *Mycobacterium smegmatis* [40]; and *Methylobacterium extorquens* DSM13060, an intracellular meristem endophyte of scots pine (*Pinus sylvestris* L.), whose defensin-like antimicrobial peptide MB1533 showed an inhibitory effect on *S. aureus* and *B. subtilis* [37]. Moreover, a similar effect has already been described for other bacterial extracts, such as *Delftia tsuruhantensus* extract, which showed antibiofilm activity against *P. aeruginosa* [42], or even some mycobacteria, such as *M. avium*, which can produce lactonase and degrade quorum-sensing autoinducers of Proteobacteria [43].

This anti-mycobacterial effect may be due to at least three non-exclusive causes: (1) the effect of DNaK and trigger factor, (2) the reactive oxygen species (ROS) generated by the xanthine oxidase and (3) the “hijacking” of pyridoxine by the xanthine oxidase. All remaining majority proteins (translation initiation factor 2, a 30S ribosomal protein S1, and the elongation factor G) and minority proteins were not considered because they used to be intimately linked with the ribosomal complex [44,45,46] or cytoplasmatic proteins, respectively. Firstly, we hypothesize that DNaK and trigger factor may link to *M. chimaera* ECMO surface and block some proteinic receptors related with intercellular communication, since the clients of these two chaperones are enriched proteins with low intrinsic solubility, proteins that tend to be members of hetero-oligomeric complexes and/or proteins that show a high density of hydrophobic patches flanked by positive [47], similar to those proteins which may be found in the non-tuberculous mycobacteria walls that at the same time are surrounded by a lipid-rich outer membrane that make them intrinsically hydrophobic and impermeable [48]. This would back up our hypothesis of the quorum quenching-like effect which *Methylobacterium* extract might exert on *M. chimaera* ECMO. Secondly, xanthine oxidase molybdopterin binding subunit might be surely result of conversion from the xanthine dehydrogenase molybdopterin binding subunit form, a very common type of enzyme found in *Methylobacterium* genus [49], to the oxidase form irreversibly by proteolysis or reversibly through oxidation of sulphydryl groups [50]. Most of these kinds of xanthine oxidases have the capability of reducing molecular oxygen and producing superoxide radical anion and hydrogen peroxide at proportions that depend on the substrate and oxidation conditions [51]. In our experimental conditions, the presence of gaseous carbon dioxide would favor the bicarbonate generation [52], and this bicarbonate would favor the superoxide radical anion [51]. This ROS may have an inhibitory effect on the *M. chimaera* ECMO due to its susceptibility to them [53]. Thirdly, it has been reported that the pyridoxine present in the growth broth used inhibits the action of certain xanthine oxidases, e.g., human xanthine oxidase [54]. This “hijacking” by the xanthine oxidase would reduce the concentration of pyridoxine, which in turn would reduce the mycobacterial growth rate because it is necessary for the correct growth of certain mycobacteria [55].

After the alarm provoked by the increasing number of *M. chimaera* infection cases associated with 3T HCD extracorporeal oxygenator, the manufacturer modified the indications for equipment disinfection, such as the time intervals of water change in the circuit, the time required between disinfection procedures, and the type of disinfectants to be used [8,29]. However, these changes were insufficient to completely eliminate the *M. chimaera* biofilm, and new cases of infection continued to appear [8,29]. Here, we demonstrate that a short dehydration period (four or five days) at room temperature followed by a 15-min treatment of *Methylobacterium* extract can reduce between 79% and 90% the *M. chimaera* biofilm adhered to a plastic surface. This treatment could be used before disinfecting contaminated equipment. In this same line of thought, Falkinham et al. [56] have recently proposed the use of a combination of enzymes, detergents and bleach as disinfestation treatment, since its use delays the reappearance of *M. chimaera*. It should be noted that the number of mycobacteria per area decreased between 96 and 120 h in the control condition, just at the same times when the extract was effective. Though it is known that most mycobacteria resist destruction for long periods in the dry state in the absence of sunlight [57] and that non-homologous end-joining pathway is involved in this phenomenon at least in *Mycobacterium smegmatis* [58], this does not mean that mycobacterial biofilms do not reduce their bacterial concentration over time at room temperature. This viability reduction may be result of an autolytic mechanism of certain mycobacteria from biofilm which would immolate themselves for the sake of their adjacent congeners [40,59], at the same time that they would weaken structurally their biofilm and make it more permeable [59,60] to the *Methylobacterium* extract and its antimycobacterial effect. These results might support the use of this extract with antibiotics or antiseptics after desiccation period of the equipment, since the *Methylobacterium* extract used in combination with clarithromycin was able to inhibit the *M. abcessus* biofilm development over time by reducing the biofilm covered area and its thickness compared to the clarithromycin control [61].

This work is not exempt from some limitations. Firstly, the results of this study can be only strictly applied to the *M. chimaera* ECMO strain isolated from a 3T HCD extracorporeal oxygenator. Despite of its clinical importance for being directly related to this concrete outbreak, the antibacterial contact effect of the extract should be tested in other *M. chimaera* strains, a cocktail of strains or even other *Mycobacterium* species. Moreover, this *Methylobacterium* extract has demonstrated to have diverse effects in different rapidly growing non-tuberculous mycobacteria [33,62]. Therefore, this study should be taken as exploratory in nature. Secondly, the antimycobacterial effect has been proven in laboratory conditions, in a surface different from the surface where this strain was isolated. For that, further investigations with this extract should be performed on other surfaces, as well as by using other biofilm development methodologies, because the molecules attached on each kind of surface may be different depending on the surface nature. Thirdly, it would be necessary to provide a proof-of-concept associated with the proteins identified separately or in combination. Furthermore, it should be ruled out that proteins smaller than those identified in this study were involved in the antimycobacterial effect of the extract. Fourthly, the use of this *Methylobacterium* extract would be uniquely limited to inert surface.

## 4. Materials and Methods

### 4.1. Bacterial Strains and Culture Conditions

We used a strain of *Mycobacterium chimaera* isolated from a 3T HCD extracorporeal oxygenator membrane (LivaNova, London) (*M. chimaera* ECMO). The strain was kept frozen at −80 °C until the experiments were performed.

*M. chimaera* was grown on Middlebrook 7H10 agar (BD, Franklin Lakes, NJ, USA) culture plates for at least five days. Before each experiment, 0.5 mL of a 2.00 ± 0.02 McFarland suspension of *M. chimaera* ECMO made in 0.9% saline was inoculated in a Bact/Alert MP bottle (Biomérieux, Îlle de France, France) supplemented with 0.5 mL of antibiotic supplement following manufacturer instructions. The suspension was then incubated for at least 120 h at 37 °C and 5% CO_2_ atmosphere [33].

### 4.2. Methylobacterium sp. CECT 7180 Extract Elaboration

Following the methodology previously described by García-Coca et al. [33], a 4-McFarland suspension of *Methylobacterium* sp. CECT 7180 from Spanish Type Culture Collection (Colección Española de Cultivos Tipo, CECT) was made in phosphate buffer saline (PBS) (Biomérieux, Îlle de France, France). This suspension was sonicated, using a Ultrasons-FB 15053 low-power bath sonicator (Thermo Fisher Scientific, Millersburg, PA, USA), three times for 30 s in ice at 100 Amp, leaving 5 min between each sonication. It was then centrifuged at 5000× *g* at 4 °C for 10 min (Thermo Scientific™ Sorvall™ ST 16 Centrifuge). Finally, the supernatant was stored and kept at −25 °C until use.

### 4.3. M. chimaera ECMO Adherence Study

For the study of the adherence of *M. chimaera* ECMO, the protocol previously described by García-Coca et al. [33], with some modifications, was employed as follow. Bact/Alert MP flasks inoculated for 120 h were centrifuged at 1160× *g* for 10 min (Thermo Scientific™ Sorvall™ ST 16 Centrifuge) and washed three times with PBS. A 0.5 McFarland suspension was made with the pellet in PBS. At the same time, four wells from a six-well plate were treated at room temperature for 15 min: two with 1 mL of PBS (control) and the other two with 1 mL of *Methylobacterium sp*. CECT 7180 extract. After 15 min, the supernatant was removed, and each well was washed once with PBS. Next, 1 mL of the 0.5 McFarland suspension was added to each well and the plate was incubated at 37 °C and 5% CO_2_ atmosphere for 90 min. The supernatant was removed, and all wells were washed once with PBS. Three ml of PBS were added to each well and the plate was sonicated using a Ultrasons-FB 15,053 low-power bath sonicator (Thermo Fisher Scientific, Pennsylvania, USA) at room temperature for 5 min. Finally, the bacterial concentration by surface area was quantified using the drop plate method [63] in Middlebrook 7H10 agar plates that were incubated for at least 10 days. This experiment was performed in triplicate.

### 4.4. Inhibition of M. chimaera ECMO Biofilm Formation

For the study of the formation of *M. chimaera* ECMO biofilm, the protocol described by García-Coca et al. [33], with some modifications, was employed as follow. After performing the adherence protocol described above on each condition, the supernatant was removed, and the wells were washed once with PBS before adding 5 mL of Middlebrook 7H9 broth (BD, New Jersey, USA) in each well. The plate was incubated for 120 h at 37 °C and 5% CO_2_ atmosphere. After this incubation, the medium was removed, and each well was washed once with PBS. Next, biofilms formed at the bottom of the well were scraped with sterile wood sticks inserted in a 50-mL Falcon tube (Falcon, Corning, Corning, NY, USA) filled with 10 mL (control surface) or 5 mL (treated surface) of PBS. The wooden sticks were sonicated for 5 min at room temperature. The bacterial concentration was quantified by means of the drop plate method [63] in Middlebrook 7H10 agar plates incubated for at least 10 days. This experiment was performed in triplicate.

### 4.5. Formation of M. chimaera ECMO Biofilm

The effect of the *Methylobacterium sp*. CECT 7180 extract on *M. chimaera* ECMO biofilm was evaluated at 24, 48, 72, 96, and 120 h using hydrophobic uncoated sterile slide 2 × 4-well plates (Ibidi GmbH, Martinsried, Germany) according to the methodology previously described by García-Coca et al. [33] and Muñoz-Egea et al. [39]. For each time point, one well was treated with 300 µL of PBS (control surface), and another well was treated with the same volume of the *Methylobacterium* extract (treated surface). A *M. chimaera* ECMO 0.5 McFarland suspension was made and 300 µL was inoculated in each well. Inoculated wells were incubated for 30 min at 37 °C and 5% CO_2_ atmosphere. The supernatant was removed, and each well was washed once with PBS. Then, 300 µL of Middlebrook 7H9 broth was added to each well, and the plate was incubated in an orbital shaker (80 rpm) at 37 °C for each development time. After each time, wells were washed once with 300 µL PBS. The wells were then stained using the Live/Dead BacLight^TM^ Bacterial Viability Kit for microscopy (Thermo Fisher Scientific, PA, USA) and the Nile Red stain (Sigma-Aldrich Co., St. Louis, MO, USA). Stains were performed according to the instructions provided by the manufacturer. After staining, plates were analyzed using a Leica DM IRB confocal laser-scanning microscope (Leica, Wetzlar, Germany): one set of wells was used to study both relative autofluorescence and Nile Red stain (covered surface), and the other was used to analyze the percentage of live mycobacteria. Each situation was studied by taking 10 random microphotographs for each stain and time set, and they were analyzed using ImageJ software (National Institutes of Health, Bethesda, MD, USA). Biofilm thickness was measured at 10 random points per well and relative autofluorescence was measured as the coefficient resulting from dividing the percentage of relative autofluorescence of covered surface by the percentage of Nile Red covered surface. This experiment was performed in triplicate.

### 4.6. Desiccation Resistance of M. chimaera ECMO Biofilms

For the study of the desiccation of *M. chimaera* ECMO biofilms, a modification of the protocol described by García-Coca et al. [33] was used. The previous protocol used on an untreated surface was performed to create 120-h *M. chimaera* ECMO biofilms in four wells of a six-well plate. The biofilms formed were left to dry at room temperature for 24, 48, 72, 96, or 120 h. After each time of dehydration, two wells were treated with 1 mL of PBS and the other two with 1 mL of *Methylobacterium sp*. CECT 7180 extract for 15 min; each treatment was performed in duplicate. Then, each well was washed once with PBS, biofilms were scraped with sterile wood sticks, and the previous protocol was applied. This experiment was performed in triplicate.

### 4.7. Statistical Analysis

Statistical analysis was performed using Stata statistical software, Release 11 (StataCrop 2009). We used a non-parametric Wilcoxon test to compare two data sets and a non-parametric Kruskal-Wallis test when more than two data sets were being compared. Statistical significance was set at *p* < 0.05. Values are provided as median and interquartile range.

### 4.8. Study of Adhered Proteins of Methylobacterium sp. CECT 7180 Extract

Two wells from a six-well plate (Fisher Scientific, PA, USA) were treated at room temperature for 15 min: two with 1 mL of PBS (control) and the other two with 1 mL of *Methylobacterium sp.* CECT 7180 extract. After 15 min, the supernatant was removed; one of the wells of each condition was washed once whilst the other one was washed twice with PBS.

The adhered proteins on each well were detached by using a modified methodology previously described by Conesa-Buendía et al. [64]. Briefly, 200 µL of RIPA buffer containing protease/phosphatase inhibitors (Sigma-Aldrich Co., St. Louis, MO, USA) and LB buffer (Thermo Fisher Scientific, Massachusetts, USA) for denaturalization were added and heated at 105 °C for 1.5 min. The supernatant with detached proteins was collected and store at −25 °C before to be used. Protein concentration was determined using bicinchoninic acid (Thermo Fisher Scientific, Massachusetts, USA). Twenty-five microliters (approximately 3 μg of protein per sample) of the detached protein were run in a 6% SDS-polyacrilamide gel. Silver staining was performed as previously described [56]. Briefly, after fixing in 50% methanol plus 5% acetic acid for 20 min and 50% methanol for 10 min, the gel was washed with ultrapure water overnight. The gel was sensitized in 0.02% Na_2_S_2_O_3_ for 1 min and then stained with 0.1% AgNO_3_ for 20 min at 4 °C. After the gel was rinsed with water, bands development was done in 0.04% formalin and 2% Na_2_CO_3_. The development was stopped with 5% acetic acid and bands were cut out and store in ultrapure water until analysis [56].

The protein bands were excised, cut into cubes (1 mm^2^), deposited in 96-well plates and processed automatically in a Proteineer DP (Bruker Daltonics, Bremen, Germany). The digestion protocol used was based on Schevchenko et al. [56] with minor variations: gel plugs were washed firstly with 50 mM ammonium bicarbonate and secondly with ACN (acetonitrile) prior to reduction with 10 mM DTT (dithiothreitol) in 25 mM ammonium bicarbonate solution, and alkylation was carried out with 55 mM IAA in 50 mM ammonium bicarbonate solution. Gel pieces were then rinsed firstly with 50 mM ammonium bicarbonate and secondly with ACN, and then were dried under a stream of nitrogen. Proteomics Grade Trypsin (Sigma-Aldrich Co., St. Louis, MO, USA) at a final concentration of 16 ng/μL in 25% ACN/50 mM ammonium bicarbonate solution was added and the digestion took place at 37 °C for 4 h. The reaction was stopped by adding 50%ACN/0.5%TFA for peptide extraction. The tryptic eluted peptides were dried by speed-vacuum centrifugation [65].

Half of each digested sample was subjected to 1D-nano LC ESI-MS/MS analysis using a nano liquid chromatography system (Eksigent Technologies nanoLC Ultra 1D plus, SCIEX, Foster City, CA) coupled to high speed Triple TOF 5600 mass spectrometer (SCIEX, Foster City, CA) with a Nanospray III source. The analytical column used was a silica-based reversed phase Acquity UPLC M-Class Peptide BEH C18 Column, 75 µm × 150 mm, 1.7 µm particle sizes and 130 Å pore size (waters). The trap column was a C18 Acclaim PepMapTM 100 (Thermo Scientific), 100 µm × 2 cm, 5 µm particle diameter, 100 Å pore size, switched on-line with the analytical column. The loading pump delivered a solution of 0.1% formic acid in water at 2 µL/min. The nano-pump provided a flow rate of 250 nl/min and was operated under gradient elution conditions. Peptides were separated using a 40 min gradient ranging from 2% to 90% mobile phase B (mobile phase A: 2% acetonitrile, 0.1% formic acid; mobile phase B: 100% acetonitrile, 0.1% formic acid). Injection volume was 5 µL [65].

Data acquisition was performed with a TripleTOF 5600 System (SCIEX, Foster City, CA). Data was acquired using an ionspray voltage floating (ISVF) 2300 V, curtain gas (CUR) 35, interface heater temperature (IHT) 150, ion source gas 1 (GS1) 25 and declustering potential (DP) 100 V. All data was acquired using information-dependent acquisition (IDA) mode with Analyst TF 1.7 software (SCIEX, USA). For IDA parameters, 0.25 s MS survey scan in the mass range of 350–1250 Da were followed by 35 MS/MS scans of 100 ms in the mass range of 100–1800 (total cycle time: 4 s). Switching criteria were set to ions greater than mass to charge ratio (m/z) 350 and smaller than m/z 1250 with a charge state of two to five and an abundance threshold of more than 90 counts (cps). Former target ions were excluded for 15 s. IDA rolling collision energy (CE) parameters script was used for automatically controlling the CE.

Mass spectrometry data obtained were processed using PeakView v2.2 Software (SCIEX) and exported as mgf files which were searched using Mascot Server v2.7.0.1 (Matrix Science, London, UK) against *Methylobacterium sp.* protein database from Uniprot (last update: 20200608, 257.559 sequences), together with commonly occurring contaminants. Search parameters were set as follows: enzyme, trypsin; allowed missed cleavages, 2; carbamidomethyl (C) as fixed modification and acetyl (Protein N-term), pyrrolidone from E, pyrrolidone from Q and Oxidation (M) as variable modifications. Peptide mass tolerance was set to ±25 ppm for precursors and 0.05 Da for fragments masses. The confidence interval for protein identification was set to ≥95% (*p* < 0.05) and only peptides with an individual ion score above 30 were considered correctly identified [65]. All reagents used were acquired in Sigma-Aldrich (Sigma-Aldrich Co., St. Louis, MO, USA).

## 5. Conclusions

In conclusion, exposing a surface to the *Methylobacterium sp*. extract inhibits *M. chimaera* ECMO biofilm development. Furthermore, this effect could be result of the effect of certain proteins, such as DNaK, trigger factor, and xanthine oxidase. This extract could be used as a pre-treatment prior to disinfection protocols for equipment contaminated with mycobacteria.

## Figures and Tables

**Table 1:** Proteins identified in each gel band by Liquid Chromatography Electrospray Ionization Tandem Mass Spectrometric (LC ESI-MS/MS).

Band	UniProt ID	Peptide Sequences	Protein Name	Species	Score ^a^ (*p* < 0.05)
1	A0A512JN39	K.LAAEDPSFR.V K.LAAEDPSFR.V K.LAAEDPSFR.V	Elongation factor G	*Methylobacterium gnaphalii*	82
	A0A2R4WM33	R.GSRATVSLPR.A	Glutathione-dependent formaldehyde dehydrogenase	*Methylobacterium currus*	50
	A0A5A8ABH1	R.AEFAESAR.A	Uncharacterized protein	*Methylobacterium sp*. P1-11	37
	A0A0X1SN19	K.TLEDLR.D	Glycerol kinase	*Methylobacterium sp.* DM1	37
	A0A2R4WQJ0	R.VALANQR.Q	SLBB domain-containing protein	*Methylobacterium currus*	31
2	A0A389MW96	R.LDSLDQR.V K.AIGAPAEAGR.- R.SGFAGSTPAGSAR.G R.AGLPYADSLTALR.G R.AAAASAAAGLSADAFK.A R.QAADAGKAEAQEAAR.A	Uncharacterized protein (translation initiation factor 2) ^b^	*Methylobacterium sp.*	198
	A0A2U8WMK9	K.VIENAEGAR.T R.TTDLMQASMK.L R.TTPSIVAFTDDGER.L	Chaperone protein DnaK	*Methylobacterium terrae*	98
	A0A0X1SM13	R.GSRATVSLPR.A	Glutamine amidotransferase	*Methylobacteriumsp.* AMS5	50
	A0A0J6SME0	R.SQVDIRPVR.D R.AQVLDVDVEKER.I	30S ribosomal protein S1	*Methylobacterium tarhaniae*	47
	A0A1E4DI60	M.IDSELR.R	4-hydroxy-tetrahydrodipicolinate synthase	*Methylobacterium sp.* SCN 67-24	43
	A0A2R4WQJ0	R.VALANQR.Q	SLBB domain-containing protein	*Methylobacterium currus*	43
	A0A0X1SN19	K.TLEDLR.D	Glycerol kinase	*Methylobacterium sp.* AMS5	41
	A0A0C6EZC1	R.EAGEVLR.G	Glycosyl transferase	*Methylobacterium aquaticum*	39
	A0A389MNS2	R.TEFAPADAK.L R.AVPGVVDVVR.I	Oxidoreductase (xanthine oxidase family protein molybdopterin-binding subunit) ^b^	*Methylobacterium sp.*	38
	A0A0C6F942	R.FSVLSR.L	Serine/threonine protein kinase	*Methylobacterium aquaticum*	38
	B0ULW9	R.LLIDVK.E	Glycosyl transferase group 1	*Methylobacterium sp.* (strain 4-46)	37
	B0UGC4	R.LERELSEARR.K	Alanine--tRNA ligase	*Methylobacterium sp.* (strain 4-46)	37
3	A0A389MRA9	K.IVADNNLK.L K.AMGGDLEAQSR.R R.SVMADVLQNAVNEANQK.I	Trigger factor	*Methylobacterium sp.*	135
	A0A0X1SM13	R.GSRATVSLPR.A	Glutamine amidotransferase	*Methylobacterium sp*. AMS5	54
	A0A0Q4WX57	R.VLSELGTR.A	Chaperone protein HtpG	*Methylobacterium sp.* Leaf91	41
	A0A2V3TYL6	R.RDISVTNPSR.R	Small-conductance mechanosensitive channel	*Methylobacterium sp.* B4	32

^a^ Ion score is −10*Log (P), where P is the probability that the observed match is a random event. Individual ion scores >16 indicated identity or extensive homology (*p*-value < 0.05). Proteins with two or more peptides were considered majoritarian in each band and are in bold. ^b^ Protein identification by Basic Local Alignment Search Tool (BLAST) according to UniProt sequence.
